# High Prevalence of Diabetic Retinopathy in Diabetic Patients Concomitant with Metabolic Syndrome

**DOI:** 10.1371/journal.pone.0145293

**Published:** 2016-01-08

**Authors:** Lu Gao, Zhong Xin, Ming-Xia Yuan, Xi Cao, Jian-Ping Feng, Jing Shi, Xiao-Rong Zhu, Jin-Kui Yang

**Affiliations:** 1 Department of Endocrinology, Beijing Tongren Hospital, Capital Medical University, Beijing, China; 2 Department of Geriatrics, Beijing Tongren Hospital, Capital Medical University, Beijing, China; 3 Beijing Key Laboratory of Diabetes Research and Care, Beijing, China; Medical University of South Carolina, UNITED STATES

## Abstract

**Objective:**

To evaluate the relationship between metabolic syndrome (MetS) and the prevalence of diabetic retinopathy (DR).

**Research Design and Methods:**

We conducted a case-controlled study, with data obtained from 2,551 Chinese participants between 18–79 years of age (representing a population of 1,660,500 in a district of Beijing). 74 cases of DR were found following data assessment by two 45° digital retinal images. Subjects without DR (NDR group) selected from the remaining 2,477 subjects were matched 1:1 to the DR group by HbA1c. MetS was defined by incorporating diagnostic criteria of the American Heart Association/National Heart, Lung, and Blood Institute (AHA/NHLBI) and the International Diabetes Federation (IDF).

**Results:**

There were no statistical differences between the DR group and NDR group in a number of biological or laboratory tests. However, the percentage of patients with DR increased vs. patients without DR with the number of MetS components from 1 to 5 (14.3% vs. 85.7%, 38.9% vs. 61.1%, 49.1% vs. 50.9%, 61.4% vs. 38.6% and 83.3% vs. 16.7%, respectively) (Pearson χ^2^ = 9.938, P = 0.037). The trend to develop DR with MetS was significantly higher than that without MetS (NMetS) (χ^2^ = 5.540, P = 0.019). MetS was an independent statistical indicator of the presence of DR after adjusting for age and sex [odds ratio (95% CI): 2.701(1.248–5.849), P = 0.012], which is still the case with an additional adjustment for WC, SBP, TC, HbA1c and duration of diabetes [odds ratio (95% CI): 2.948(1.134–7.664), P = 0.027].

**Conclusion:**

DR is one of the diabetic microvascular complications. Apart from poor glycemic control, the concomitance of other metabolic factors can also influence DR. MetS, defined as a cluster of metabolic risk factors, is a strong and independent indicator of DR, even to the same extent as glycemic control.

## Introduction

Diabetic retinopathy (DR) is one of the most common and severe microvascular complications of diabetes mellitus (DM), and the leading cause of irreversible blindness. The prevalence of DR is expected to increase, and the number of people at risk of vision loss is predicted to double by the year 2030 with the increasing rate of diabetes epidemic [1]. It is now known that duration of diabetes and degree of hyperglycemia play a key role in DR, but maintenance of normoglycemia does not always completely prevent the development of DR. Thus, additional factors related to the diabetic state are postulated to have a causal role in this disorder.

Metabolic syndrome (MetS), defined as a cluster of cardiovascular risk factors including impaired glucose regulation, central obesity, dyslipidemia and hypertension, is known to be associated with pathological changes in blood vessels. In China, individuals with MetS are three to ten times more likely to develop cardiovascular disease [2]. In addition to large-vessel disease, there is increasing evidence that MetS may also affect the microvascular disorders [3, 4].

Many of the metabolic risk factors, and even the overall concept of MetS itself, have sparked a lot of debate regarding their precise roles on DR. Moreover, most previous research was conducted in the west and did not include results specific to the Chinese population. The aim of this study was to evaluate the relationship between the diagnosis of MetS and the prevalence of DR in diabetic and pre-diabetic Chinese people. To avoid any strong influence on DR by hyperglycemia, we conducted a case-control study with matching HbA1c level.

## Research Design and Methods

### Study population

Between July 2010 and March 2011, the Health Examination Survey, a cross-sectional, population-based survey on chronic diseases and risk factors was conducted in Changping, one of the suburban districts in Beijing with an area of 1,343.5 square kilometers and a permanent population of 1,660,500. Household sampling was performed by the Center for Disease Control and Prevention (CDC) of Beijing; 8,155 randomly selected households were eligible (occupants were of Chinese nationality and had lived in the district for at least 6 months). All residents 18–79 years of age were enumerated in each sampled household; then, using Kish’s selection tables [5], one person was randomly chosen to participate in the study. The sample was chosen irrespective of availability at home during the field visit and medical history of diabetes. Of the 8,155 individuals, 8,084 received baseline examinations including a physical examination, fasting plasma glucose (FPG) measurements, and renal and liver function tests in addition to completing a general health questionnaire. Next, the 3,760 subjects whose FPG≥ 5.6 mmol/l were invited to perform a 75g oral glucose tolerance test (OGTT) and ophthalmic examination by Beijing Diabetes Prevention and Treatment Office and field workers, and 2,592 (68.9%) agreed to participate in the study. After excluding 31 subjects who had cataracts, 6 with glaucoma and 4 with other eye diseases, a total of 2,551 individuals successfully completed the OGTT and ophthalmic examination, with 74 participants being diagnosed with DR. NDR group was matched 1:1 to DR group by HbA1c, fasting status and time of blood draw. Thus, 74 DR patients and 74 HbA1c- matched NDR subjects comprised the study groups.

### Data collection

All participants received a standardized examination. Data regarding age, smoking, alcohol consumption and history of diabetes and hypertension were obtained with a standardized questionnaire. Anthropometric measurements were taken with each subject wearing light clothing and without shoes. Body Mass Index (BMI) was calculated as weight (kg) divided by height squared (m^2^). Waist circumference (WC) was measured at the level of the umbilicus in cm. Blood pressure (BP) was measured 3 times when participants were seated, and the average of the last 2 measurements was adopted. Blood samples were collected after an overnight fast for the determination of plasma glucose, HbA1c, total cholesterol (TC), triglycerides (TG), high density lipoprotein cholesterol (HDL-C) and low density lipoprotein cholesterol (LDL-C) concentrations, followed by an OGTT between 08:00 and 10:00 hours. Subsequently, another blood sample was obtained to determine post-loading plasma glucose levels. These specimens were analyzed within 24 hours.

### Definition of clinical and biochemical variables

Central obesity is based on WC cutoff ≥ 80 cm in women and ≥ 90 cm in men according to the IDF ethnicity-specific definitions for Asian [6]. Overweight and obesity are respectively identified as BMI 24–28 kg/m^2^ and BMI equal to or over 28 kg/m^2^ according to the Working Group on Obesity in China (WGOC) (2002) [7]. We defined BMI ≥24 kg/m^2^ as general obesity in our study. Cutoff for hypertension is blood pressure ≥140/ ≥90 mmHg [8]. Patients on anti-hypertensive drugs are assumed to be hypertensive. The cutoff for diabetes is glucose level ≥7 mmol/l for fasting and ≥11.1 mmol/l for 2 hours (2h PG). Pre-diabetes was defined as FPG 5.6~6.9 mmol/l and/or in the OGTT 2h PG 7.8~11.0 mmol/l [9].

### Definition of MetS

According to the consensus definition (incorporating the International Diabetes Federation (IDF) and the American Heart Association/National Heart, Lung, and Blood Institute (AHA/NHLBI) definitions) [6], metabolic syndrome is diagnosed when three or more of the following risk factors are present: a. abdominal obesity (WC ≥80 cm in women and ≥90 cm in men); b. SBP ≥130 mmHg or DBP ≥85 mmHg on examination or when the participant had a prior diagnosis of hypertension and was receiving specific medication; c. level of TG ≥150 mg/dl (1.7 mmol/l) or specific medication; d. low HDL-C: <40 mg/dl (1.0 mmol/l) in males, <50 mg/dl (1.3 mmol/l) in females or specific medication; and e. FPG ≥100 mg/dl (5.6 mmol/l) or history of diabetes mellitus or taking antidiabetic medications. In our study, we also defined criteria b, c and d as impaired BP, impaired TG and impaired HDL-C, respectively.

### Assessment of retinopathy

All participants who underwent OGTTs received eye examinations by an ophthalmologist and had a bilateral retinal photograph taken of the fundus through dilated pupils. Two 45u color digital images of the retina were taken of each eye by a technologist using a Topcon TRC-NW7SF fundus camera (Topcon, Tokyo, Japan), an ophthalmic digital imaging system. The first image was centered on the macula, and the second on the optic nerve. The photographs were graded by two qualified ophthalmologists from the Eye Center of Capital Medical University, Beijing Tongren Hospital according to the international clinical diabetic retinopathy severity scale [10]: (i) no retinopathic changes (NDR); (ii) mild non-proliferative retinopathy (NPDR); (iii) moderate NPDR; (iv) severe NPDR; and (v) proliferative retinopathy (PDR). The degree of DR was determined according to the grading in the most affected eye. The ophthalmologists grading the photographs were blinded to the patients’ characteristics.

### Statistical analysis

All statistical analyses were conducted with the software package SPSS version 18.0 for Windows. For the continuous variables with a normal distribution, mean±SD was reported and the independent t-test was used to compare subjects with no diabetic retinopathy (NDR) with any stage of diabetic retinopathy (DR). For the discrete variables or the continuous variables without a normal distribution, the median (P25–P75) was reported, and a Mann-Whitney rank test was used to examine the differences between the groups. In the meantime, distribution of discrete/qualitative variables was compared by Pearson chi-square test. Multivariable logistic regression analysis was used to estimate crude and adjusted odds ratios (ORs) (95% CIs) to allow for differences between groups with respect to demographic and risk factors and control for potentially confounding variables. A p-value of less than 0.05 was considered statistically significant.

### Ethics statement

The study was conducted with the approval from the Ethics Committee of Beijing Tongren Hospital, Capital Medical University. Written informed consent was obtained from each participant.

## Results

Of the 2,551 study participants, 74 (2.90%) were found to have DR. Mild NPDR, moderate NPDR, severe NPDR and PDR were found in 37 (1.45%), 28 (1.10%), 2 (0.08%) and 7 (0.27%) subjects respectively. NDR group was matched 1:1 to DR group by HbA1c, fasting status and time of blood draw.

The demographic and biochemical parameters of the two groups are shown in Table 1. There were no statistical differences between DR group and NDR group in clinical characteristics (age, sex, duration of diabetes, levels of FPG, 2h PG, HbA1c, SBP, DBP, TC, TG, HDL-C, LDL-C, WC and BMI, and the proportion of smoking, alcohol, central obesity, generalized obesity, DM and hypertension).

**Table 1:** Clinical characteristic of studied subjects with and without diabetic retinopathy.

	NDR	DR	P-value
n	74	74	
Age (year)	55.15±7.48	54.6±8.52	0.678
Female (%)	44.6	47.3	0.741
HbA1c (%)	8.67±2.21	8.67±2.21	matching
FPG (mmol/l)	9.82(7.99, 12.69)	9.44(7.76, 13.43)	0.925
2h PG (mmol/l)	17.78±7.60	18.13±7.35	0.776
DM (%)	85.1	89.2	0.731
Duration of diabetes (years)	10.78±5.71	10.88±5.68	0.915
SBP (mmHg)	146.01±22.96	152.57±22.63	0.083
DBP (mmHg)	84.66±11.23	88.28±12.01	0.060
TC (mmol/l)	5.42(4.88, 6.01)	5.19(4.59, 6.03)	0.485
TG (mmol/l)	1.75(1.20, 2.85)	1.95(1.30, 2.80)	0.328
HDL-C (mmol/l)	1.50±0.26	1.42±0.30	0.115
LDL-C(mmol/l)	2.77±0.49	2.70±0.53	0.365
Smoking (%)	25.7	28.4	0.711
Alcohol (%)	36.5	32.4	0.604
WC (cm)	89.41±11.85	89.90±7.99	0.768
BMI (kg/m^2^)	26.01(23.98, 29.09)	26.61(24.00, 27.95)	0.913
Generalized obesity (%)	74.3	75.7	0.849
Central obesity (%)	63.5	67.6	0.604
Impaired BP (%)	71.6	83.8	0.076
Impaired TG (%)	50.0	58.1	0.322
Impaired HDL-C (%)	9.5	18.9	0.099

Data are means±SE, median (P25–P75) or raw numbers (%). Continuous data were used for univariate general linear models and categorical data were analyzed by χ^2^ tests.

Abbreviation: DR, diabetic retinopathy; NDR: non-diabetic retinopathy; WC, waist circumference; BMI, body mass index; TC, total cholesterol; TG, triglycerides; HDL-C, high- density lipoprotein; LDL-C, low-density lipoprotein cholesterol; FPG, fasting plasma glucose; 2h PG, 2-h post oral glucose load plasma glucose; HbA1c, glycated haemoglobin; SBP, systolic blood pressure; DBP, diastolic blood pressure; DM, diabetes mellitus; Impaired BP, Impaired TG and Impaired HDL-C was defined by criteria b, c and d of MetS respectively.

**Fig 1:**
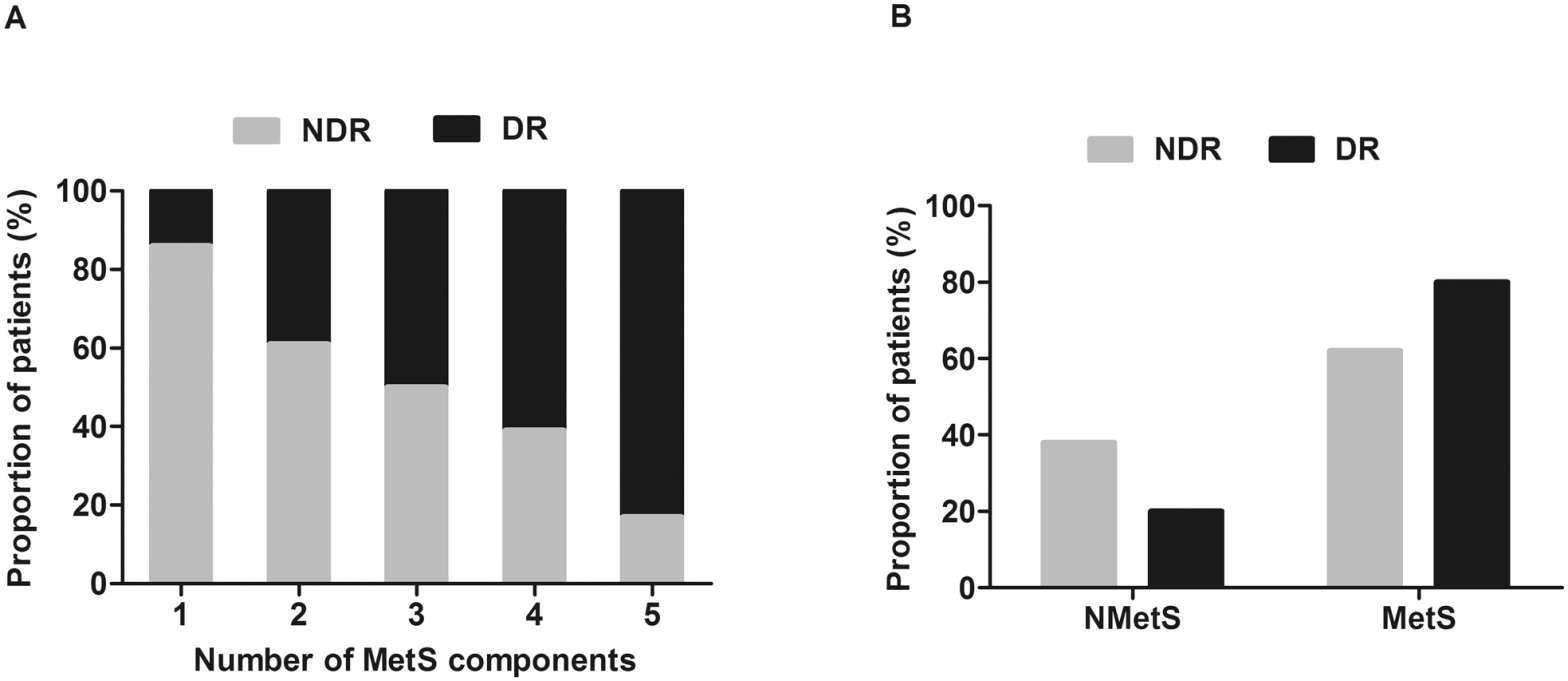
The association of diabetic retinopathy with metabolic syndrome. **(A)** Prevalence (%) of patients with DR in relation to number of metabolic syndrome components (1 to 5). 1 represents elevated glucose, while 2 to 5 represent elevated glucose combined with one to four other components of MetS, respectively. When patients were grouped according to the number of detected components of the metabolic syndrome, the percentage of patients with DR increased linearly with the increasing number of components in their respective groups (14.3%, 38.9%, 49.1%, 61.4%, 83.3%, respectively), whereas, the percentage of patients without DR decreased with the increasing number of components (85.7%, 61.1%, 50.9%, 38.6%, 16.7%, respectively) (Pearson χ^2^ = 9.938, P = 0.037). **(B)** According to the IDF and AHA/NHLBI definitions. Comparison of prevalence of DR between NMetS and MetS group. The trend to DR in the MetS group was significantly higher than in the NMetS group (Pearson χ^2^ = 5.540, P = 0.019).

**Fig 2:**
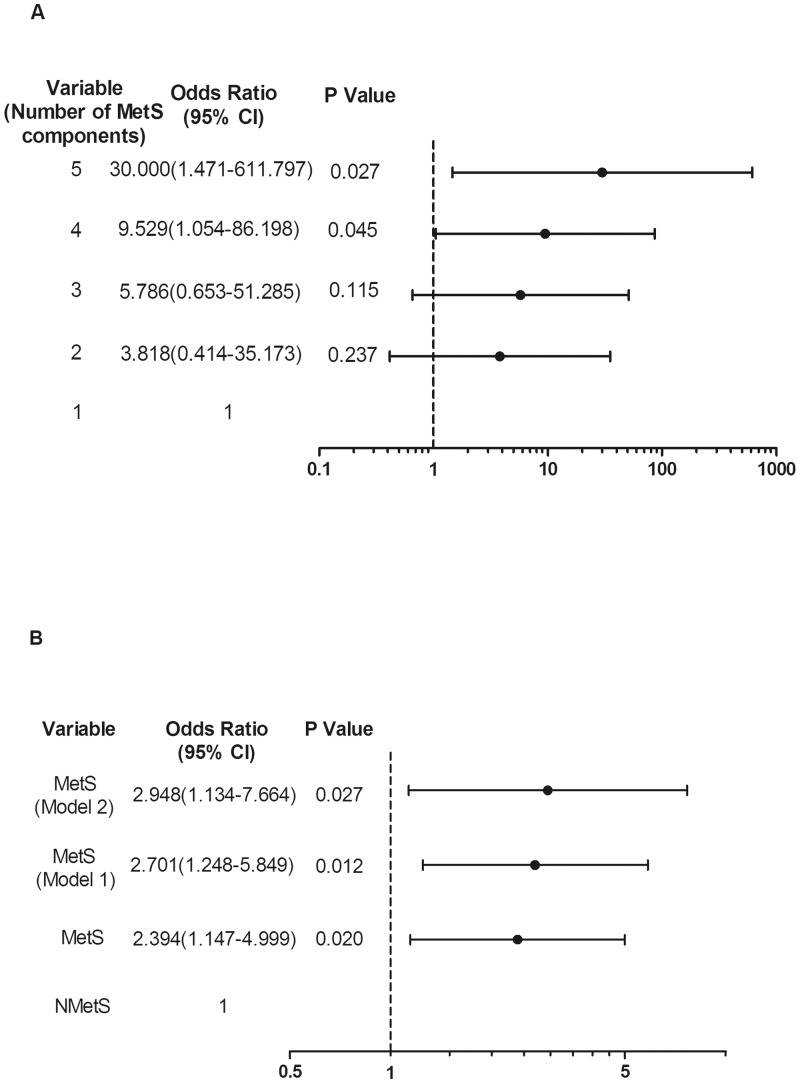
Contribution of cumulative metabolic components in DR and Odds ratios for DR with/without MetS. Binary logistic regression was conducted to assess the association of DR with the number of components of MetS (A) and MetS (B) using the Entry method; adjusted odds ratios (ORs) and the 95% confidence intervals (CIs) given. 1–5 were identified as the number of components of MetS in (A), with 1 being elevated glucose as a referent and 2–5 elevated glucose combining one to four other factors of the MetS. The group without MetS was used as a referent in (B). Adjustment variables included the basic confounders (age and sex) in Model 1. In Model 2, WC, SBP, TC, HbA1c and duration of diabetes were also considered other adjustment variables and were thus added to Model 1.

However, when patients were grouped 1–5 according to the number of detected components of the MetS (1 represented elevated glucose, while 2 to 5 represented elevated glucose combined with one to four other components of MetS, respectively), the percentage of patients with DR increased linearly with the increasing number of components in their respective groups (14.3%, 38.9%, 49.1%, 61.4%, 83.3%, respectively), and the percentage of patients without DR decreased with the increasing number of components (85.7%, 61.1%, 50.9%, 38.6%, 16.7%, respectively) (Pearson χ^2^ = 9.938, P = 0.037), as is illustrated in Fig 1A. Of the 43 subjects without MetS, 15 had DR. However, in the MetS group of 105 subjects, the number was 59.The trend to DR in MetS group was thus significantly higher than in NMetS group (χ^2^ = 5.540, P = 0.019), as is illustrated in Fig 1B. Next, we performed logistic regression analysis to identify the risk of DR with the number of components of MetS. The risk for DR was significantly elevated with the increment of MetS components. The aggregation of elevated glucose combining three or four other metabolic factors in the MetS increased 9.529 times [odds ratio (95% CI): 9.529 (1.054–86.198), P = 0.045] or 30.000 times [odds ratio (95% CI): 30.000 (1.471–611.797), P = 0.027] than those with elevated glucose only (Fig 2A).

To determine if there is an independent association between DR and MetS, we performed multivariable logistic regression analysis. With an adjustment for age and sex, MetS was significantly associated with and an independent risk factor for DR [odds ratio (95% CI): 2.701(1.248–5.849), P = 0.012]. Moreover, the association was not affected by an additional adjustment for WC, SBP, TC, HbA1c and duration of diabetes [odds ratio (95% CI): 2.948(1.134–7.664), P = 0.027] (Fig 2B).

## Discussion

DR is an important and common microvascular complication of diabetes mellitus. American Diabetes Association (ADA) reported that nearly all patients with type 1 diabetes and more than 60% of patients with type 2 diabetes (T2D) had DR after 20 years of diagnosis [11]. Different from other studies that focus on the diabetic people, the research subjects we chose were pre-diabetics and diabetics with FPG of 5.6 mmol/l or greater, which conforms to the diagnostic criteria for MetS we adopted. More importantly, the prevalence of pre-diabetics is increasing. Around 5–10% of people with this problem become diabetic every year, and current estimates indicate that up to 70% of pre-diabetic states eventually develop diabetes [12, 13]. Moreover, pre-diabetes is often under-detected and remains asymptomatic, which may elevate future risks of diabetes and cardiovascular complications [14–16]. In our study, 8 out of 74 (10.81%) DR patients were pre-diabetics.

It has already been proved that the duration of diabetes and degree of hyperglycemia are the main predictors of the prevalence and progression of DR [17–19]. However, some research has indicated that such development and progression also appeared in diabetic patients with maintenance of normoglycemia. This implies that multiple risk factors may also have an impact. For this reason, NDR group was matched 1:1 to DR group by HbA1c in our study, with the levels of FPG and 2h PG and duration of diabetes bearing no difference between the two groups so as to look for other contributing factors for DR.

Many studies have shown that in addition to elevated glucose, other biological entities such as obesity, elevated blood pressure and atherogenic dyslipidemia can also influence DR[19–21]. However, these biological entities combine to form MetS, which is defined by a constellation of interconnected physiological, biochemical, clinical, and metabolic factors that confers a 5-fold increase in the risk of type 2 diabetes mellitus (T2DM) and 2-fold the risk of developing cardiovascular disease (CVD) over the next 5 to 10 years [6]. It is considered as a first order risk factor for atherothrombotic complications and directly results in increased macrovascular events and mortality in diabetic patients. Unfortunately, some previous studies on the association of MetS and diabetic microvascular complications have registered contradictory results. The Metascreen Writing Committee reported that MetS defined by the National Cholesterol Education Program (NCEP) and IDF guidelines was a strong, independent indicator of microvascular complications in patients with type 2 diabetes [22]. However, a Japanese study revealed that MetS defined by IDF was not associated with the presence of diabetic microvascular complications [23]. In our research, no significant statistical difference was found between the NDR and DR groups regarding most metabolic indices, but the DR figures were still slightly higher than NDR ones. When we went one step further by diagnosing each component of the MetS according to the criteria issued by the consensus of AHA/NHLBI and IDF in 2009, with a sub-group designed especially for Asian subjects, we found a greater proportion of people with DR from the group with three or more MetS components (MetS group) than from NMetS group. In other words, the trend towards DR in MetS group was significantly higher than in NMetS group, which suggested that MetS was significantly associated with DR.

Concurrent metabolic alterations occur more frequently than would be expected by chance and such factors increase cardiovascular risk over and above the risk associated with the individual factors alone [24]. Of all 148 subjects in our two study groups, we found that 7 people (4.72%) had elevated glucose only, while the other 141 (95.28%) had elevated glucose combined other factors. In addition, if these hyperglycemic people were combined with two or more other factors of the MetS, the risk of DR was significantly higher than those with glycemic factor only, and it grew with the increasing number of MetS components present. As a whole, such a constellation of metabolic abnormality became highly recognizable in DR group when MetS was used as an observational parameter, which is of great statistical significance. It is therefore not surprising that apart from poor metabolic control, the combination of metabolic factors can also influence DR.

These abnormal metabolic factors have a direct impact on DR, as epidemiological data from various studies have identified that they may lead to an increase in insulin resistance, inflammatory, hypercoagulable state, oxidative stress, endothelial dysfunction, and eventually DR development [25–28]. They also worsen ineffective glucose control and hence affect DR indirectly. The relationship may be due to either greater difficulties in achieving glucose control in patients with MetS and/or an aggravating role of poor glucose control on the variables compounding the MetS. Because of the components of MetS interacting and influencing each other, if the diagnosis of the MetS is confirmed, the adverse effect on the DR by this metabolic disorder syndrome is much larger than when these metabolic factors exist alone.

Randomized clinical trials demonstrated that intensive, multifactorial interventions were effective in reducing both macrovascular and microvascular complications in type 2 diabetes patients [19]. This was manifested in our study, to help prevent DR, it is insufficient to control any one risk factor, instead intensified, targeted and multifactorial intervention aimed at several modifiable risk factors should be implemented in patients with DM.

Our study has several limitations that must be taken into account. First of all, as these associations are cross-sectional, the study design is incapable of estimating causal relation directly; therefore, our findings may suggest that MetS is an indicator, but not predictor of DR. Secondly, our study participants are selected from China and the definition of MetS in this study is proposed by AHA/NHLBI and IDF in 2009 with a sub-group designed especially for Asian subjects. Cohort studies based on different ethnic populations using different definitions are needed to confirm the adverse effects of MetS on DR. Last but not least, we have no further classification of DR, so we cannot clarify which level of DR is most influenced by MetS. However, we have no reasons to believe these would substantially bias the associations reported herein.

In conclusion, all our data show that patients with multiple risk factors are more likely to develop DR. As a cluster of metabolic risk factors, MetS exists as a meaningful pathological process. It is closely associated with and is a strong, independent indicator of DR. A timely diagnosis of the MetS provides valuable information regarding the risk of DR, and should therefore be added to the checklist of diabetic microvascular complications prevention mechanisms.

## Supporting Information

S1 Data Relevant data underlying the findings described in manuscript. (XLS)
